# Diabetic retinopathy screening in incident diabetes mellitus type 2 in Germany between 2004 and 2013 - A prospective cohort study based on health claims data

**DOI:** 10.1371/journal.pone.0195426

**Published:** 2018-04-05

**Authors:** Daniel Kreft, Myra B. McGuinness, Gabriele Doblhammer, Robert P. Finger

**Affiliations:** 1 Rostock Center for the Study of Demographic Change, Rostock, Germany; 2 Empirical Methods in Social Sciences and Demography, Institute for Sociology and Demography, University of Rostock, Rostock, Germany; 3 Centre for Eye Research Australia, Royal Victorian Eye and Ear Hospital, East Melbourne, Australia; 4 Ophthalmology, Department of Surgery, University of Melbourne, Melbourne, Australia; 5 German Center for Neurodegenerative Diseases (DZNE), Bonn, Germany; 6 Ophthalmic Epidemiology, Department of Ophthalmology, University of Bonn, Bonn, Germany; Soochow University Medical College, CHINA

## Abstract

**Objective:**

To assess factors associated with diabetic retinopathy (DR) screening uptake following a diagnosis of type 2 diabetes mellitus (type 2 diabetes) in Germany.

**Materials and methods:**

A nationally representative prospective sample of individual-level health claims data for 250,000 members from Germany’s largest public insurance provider in 2004–2013 was assessed. In the sample, 26,560 persons with incident type 2 diabetes were identified. Factors associated with subsequent DR screening were assessed using descriptive statistics, Kaplan-Meier estimator, and Cox regression analysis.

**Results:**

On average 27.6 visits to an ophthalmologist per 100 person-years in persons with incident type 2 diabetes occurred. Half of all incident cases (Kaplan-Meier estimator) had not seen an ophthalmologist after more than two years (2.25 years) following their diabetes diagnosis. In the multivariate analysis, an older age (from hazard ratio HR(70–74) = 0.93 [95%-CI: 0.89–0.97] to HR(90+) = 0.50 [95%-CI: 0.42–0.60] compared to persons aged 50–69 years) and a higher disability level (i.e. HR(disability level 3) = 0.30 [95%-CI: 0.25–0.36]) were associated with a lower likelihood, while female sex (HR = 1.12 [95%-CI: 1.08–1.15]), six or more comorbidities (HR = 1.26 [95%-CI: 1.15–1.37]), moderate (HR = 1.51 [95%-CI: 1.46–1.56]) or severe type 2 diabetes (HR = 1.53 [95%-CI: 1.45–1.61]) as well as being enrolled in a type 2 diabetes disease management program (HR = 1.78 [95%-CI: 1.69–1.87]) were associated with a higher likelihood of DR screening.

**Conclusions:**

A high proportion of newly diagnosed persons with type 2 diabetes did not follow current German recommendations for DR screening, impeding timely detection and management of potential complications. This was more apparent among persons who were men, older or had a disability. The uptake of screening was considerably greater among those enrolled in a diseases management program. These factors need to be considered when planning DR screening services and/or referrals.

## Introduction

Diabetic retinopathy (DR) is a leading cause of blindness in working-aged adults in industrialized countries [1]. As a microvascular and often insidious complication of diabetes, many people with DR remain completely asymptomatic during early stages of the disease and are unaware that their vision is under threat. Highly effective treatments to prevent visual loss due to diabetes have been developed, including timely laser photocoagulation, which can reduce severe vision loss due to DR by at least 94% [2–4], intravitreal anti-vascular endothelial growth factor (anti-VEGF) and steroid injections [5; 6]. However, the effectiveness of these treatments relies on early diagnosis of DR. Both DR screening and treatment have been found to be highly cost-effective from a healthcare payer as well as a societal perspective [7].

Almost all current diabetes mellitus type 2 (type 2 diabetes) guidelines recommend DR screening at diagnosis and at least biennially thereafter [8–11]. In Germany, annual DR screening is recommended, biannual if no risk factors for DR are present [9; 12]. Guideline-adherent diabetes care, including regular eye examinations, has been shown to considerably reduce the occurrence of low vision and blindness in persons with diabetes [13; 14]. Despite clear guidelines, however, DR screening uptake is consistently below recommended levels [15–18] and in most parts of the world diabetic screening programs remain non-systematic (i.e. not population-based and/or national) [19].

A number of factors related to the patient, provider and healthcare system impact DR screening uptake. Among the most important patient related factors are age, diabetes duration, health literacy and socio-economic status [19–23]. Important provider/healthcare system related factors are diabetes disease management programs (DMPs) or similarly structured programs such as national DR screening programs which have been demonstrated to not only lead to better disease control but also to increased DR screening uptake [24; 25]. As type 2 diabetes is a chronic, slowly progressing disease, large longitudinal studies are required to sufficiently assess these factors and how they are inter-related. To date, most studies fall short on this, being too small and/or not following persons long enough [20–22].

Against this background we investigated factors associated with the uptake of DR screening in incident type 2 diabetes over ten years in a population-based sample in Germany.

## Material and methods

### Data

Members of the largest German public health insurance provider, the “Allgemeine Ortskrankenkasse” (AOK), were randomly sampled for this analysis. Data from a total of 250,000 persons born prior to 1955 and living in private households and institutions was obtained. Data access was legally approved by the Scientific Institute of the AOK (WIdO). The study is based on anonymized administrative claims data that never involved persons directly. Individual persons cannot be identified, and the analyses presented do not affect persons whose anonymized records were used. Thus, no ethical approval was needed. The study complies with the tenets of the Declaration of Helsinki.

Medical individual-level data for all 250,000 members was registered and collected quarterly from the beginning of 2004 until the end of 2014, or until an earlier exit from the study due to a change of health insurance or death. The collected data covers general demographic data, inpatient and outpatient diagnosis data coded using the International Classification of Disease 10th revision (ICD-10), medical treatment data coded using the German Procedure Classification (OPS), and prescriptions of medications coded using the German Anatomical Therapeutic Chemical (ATC)-Classification. The type of healthcare provider associated with each outpatient episode was also collected.

### Population under study

Persons aged 50+ who experienced a first diagnosis of type 2 diabetes during the observation period were included. Persons with an ICD-10 code of E11 were considered to have type 2 diabetes. We used the first two quarters of 2004 as a baseline to differentiate between prevalent and incident type 2 diabetes patients: those with a type 2 diabetes diagnosis in the first half of 2004 were defined as prevalent cases assuming that the first unobserved type 2 diabetes diagnosis occurred in the time before 2004. Type 2 diabetes diagnoses after the first half of 2004 were defined as incident type 2 diabetes (n = 34,491). Incident type 2 diabetes patients with a chronic eye disease which necessitated regular ophthalmic check-ups (i.e. any type of glaucoma [ICD-10 code H40], cataract [H26], age-related macular degeneration or other macular disease [H35.3], or retinopathy [H35.0–2]) present in the quarter before the first type 2 diabetes diagnosis were excluded (n = 7,931). In total, 26,560 persons were included in the analysis sample.

### Validation strategy

We used a validation strategy to ensure that only verified incident type 2 diabetes patients were included. We defined a type 2 diabetes diagnosis as a valid incidence if the first diagnosis was followed by at least one second "validating" diagnosis in a later quarter until the end of 2013. The same validation strategy was also applied to the diagnoses of any of the chronic eye diseases and the diseases used for the comorbidity status (see below).

### Outcome

The outcome of the analysis was the first contact with an ophthalmologist following incidence of type 2 diabetes. In Germany, DR screening is provided solely by ophthalmologists, no other medical providers such as opticians or optometrists diagnose or manage DR. Thus, only diagnoses of registered ophthalmologists in single or joint practices were considered as no other healthcare provider can screen for DR in Germany. For the analysis, the specific diagnosis recorded by the ophthalmologist was irrelevant.

### Control variables

From the data available, five factors were considered *a priori* to be potential predictors of screening uptake and were included in the multivariable models. These factors were sex, age at first diagnosis (categorized as 50–69, 70–74, 75–79, 80–85, 85–89, and 90+), comorbidity status, severity of type 2 diabetes, level of disability, and participation in a type 2 diabetes DMP.

The comorbidity status was adapted from the comorbidity index by Charlson et al. [26] and measured the total number of experienced diagnoses of selected groups of severe diseases within the observation period. These diseases were acute myocardial infarction (ICD-10 code I21-I22, I25.2), cerebrovascular diseases (G45-G46, H34.0, I6), ischemic (I20-I25) and other heart diseases (I43, I50, I09.9, I11.0, I13.0–2, I25.5, I42.0–9, P29.0), cancer (C00-C97), kidney diseases (N11-N19, I12.0, I13.1–2, N03.2–7, N05.2–7, N25-N29, Z49.0–2, Z94.0, Z99.2), lung diseases (J44), liver diseases (B18, K70, K71.1,3,4,5,7, K72.1,9, K76.0,2–9, Z94.4), nervous diseases (G0-1, G4-9, G20-22, G23.0,2,8,9, G24-26, G31.2,9, G31.81,88, G32,35–37), dementia (F00.0–9, F01.0–9, F02.0–8, F03, F05.1, G23.1, G30.0–9, G31.0 G31.82) and injuries of the lower extremities, hips and pelvis (S7-S9). All diagnoses were validated as outlined above. The resulting score was categorised into four self-defined groups: None of the severe diseases, one or two, three to five, or six and more of the diseases.

The severity of type 2 diabetes was classified according to the anti-diabetic medication dispensed by a pharmacy. Type 2 diabetes was defined as low severity if no medication was dispensed (presumably diet controlled), as moderate severity if only oral medication (ATC codes: A10BA, A10BB, A10BC, A10BD, A10BF, A10BG01, A10BG02, A10BG03, A10BP, A10BX) was dispensed, and of high severity if insulin (ATC code: A10A) was dispensed.

The disability level (Pflegestufe) was based on the officially assigned care status based on presence of formally assessed physical and/or mental disability. The assessment of disability is routinely executed by medical experts and standardised by legal definitions and measures. Three disability levels are defined by law for those with no disability, with disability level 1 as the lowest severity of limitations and disability level 3 as the most severe level.

Participation in a type 2 diabetes DMP was measured as a yes-no-variable.

In all analyses, sex and age at first type 2 diabetes diagnosis were constant attributes, while the comorbidity status, the severity of type 2 diabetes, the disability level, and the participation in a type 2 diabetes DMP may vary over the observation period.

### Statistical analysis

Unadjusted incidence rates were estimated separately for all selected control factors. Kaplan-Meier (KM) survivor functions were computed and Cox regression analysis was performed. Effect modification by sex was investigated for each of the covariates. Subsequently, sensitivity analyses were performed to evaluate the choice of explanatory variables: by excluding particular covariates, the changes in the effect size of the other covariates are observed. All analyses were performed by Stata/IC version 12.1 (College Station, TX).

## Results and discussion

### Sample characteristics

Of the 26,560 persons included in the analysis, 48.4% were men and 51.6% were women. At the quarter of the first validated type 2 diabetes diagnosis, the majority (55.3%) of the sample were between 50 and 69 years of age, with decreasing proportions in the older age categories (Table 1). Among those with incident type 2 diabetes, 22.0% were reported to have none of the selected comorbid diseases at first diagnosis, 47.1% had one or two, 27.9% had three to five, and 3.0% had six or more severe diseases. The majority (86.5%) had no legally recognised disability. Six per cent entered into a type 2 diabetes DMP in the quarter of the first type 2 diabetes diagnosis.

**Table 1:** Sample characteristics of included persons at the first type 2 diabetes diagnosis, AOK data.

Characteristics		n (N = 26.560)	%
**Sex**	Men	12,861	48.4%
** **	Women	13,699	51.6%
**Age at**	50–69	14,690	55.3%
**first diagnosis**	70–74	4,566	17.2%
** **	75–79	3,339	12.6%
** **	80–84	2,229	8.4%
** **	85–89	1,097	4.1%
** **	90+	639	2.4%
**Number of**	0	5,836	22.0%
**comorbid diseases**	1–2	12,517	47.1%
** **	3–5	7,407	27.9%
** **	6 and more	800	3.0%
**Severity of**	Low	17,919	67.5%
**type 2 diabetes**	Moderate	6,635	25.0%
** **	High	2,006	7.6%
**Disability level**	No disability	22,973	86.5%
** **	Disability level 1	1,655	6.2%
** **	Disability level 2	1,487	5.6%
** **	Disability level 3 (most severe)	445	1.7%
**Participation in a**	No	24,965	94.0%
**type 2 diabetes DMP**	Yes	1,595	6.0%

The mean follow-up time starting at the first type 2 diabetes diagnosis was 5.2 years and the median follow-up time was 5.5 years.

### Rate of first ophthalmological visit

In total, there were 16,645 persons who visited an ophthalmologist at least once during the observation period, resulting in a mean time at risk of 2.2 years and a rate of 27.6 first visits per 100 person-years in the newly type 2 diabetes diagnosed persons (95% CI: 27.2–28.0, Table 2). For men, there were 27.8 first visits [95% CI: 27.2–28.4] and 27.3 visits for women [95% CI: 26.7–27.9]. First ophthalmological visit decreased with increasing age from 31.5 visits [95% CI: 30.9–32.1] for persons aged 50–69 to 10.2 visits [95% CI: 8.6–12.3] for those aged 90+. Persons with a greater number of comorbidities were less likely to access an ophthalmologist following their type 2 diabetes diagnosis: there were 31.3 first visits for persons with no comorbidities [95% CI: 30.2–32.4], compared to 19.5 visits for persons with 6 or more comorbidities [95% CI: 18.0–21.0]. The incidence was highest in persons with moderate severity of type 2 diabetes (38.5 first visits, 95% CI: 37.4–39.6) and much lower in low (23.7 visits, 95% CI: 23.2–24.1) and high severity (32.5 visits, 95% CI: 30.9–34.2). Ophthalmologist attendance decreased from 31.3 first visits [95% CI: 30.8–31.8] for persons with no disability to 6.4 visits [95% CI: 5.3–7.7] for those with the highest level of disability. The access rate for persons in a DMP was much higher than for those not enrolled in a DMP (53.7 visits [95% CI: 51.3–56.2] and 25.9 [95% CI: 25.5–26.4] respectively).

**Table 2:** Yearly incidence rates and Kaplan-Meier estimators of time until first visit to an ophthalmologist by attributes of the type 2 diabetes patients, third quarter of 2004 to last quarter of 2013, AOK data.

Characteristics		n	Time at risk in years	Events	Incidence rate of first visit (95%-Confidence intervals)						Years from first diagnosis to first visit Kaplan-Meier estimator (in years)		
											25%	50%	75%
**Sex**	Men	12,861	28,985	8,069	0.278	(	0.272	-	0.284	)	0.75	2.25	6.25
** **	Women	13,699	30,727	8,396	0.273	(	0.267	-	0.279	)	0.75	2.00	6.75
**Age at**	50–69	14,690	33,188	10,439	0.315	(	0.309	-	0.321	)	0.50	1.75	5.00
**first diagnosis**	70–74	4,566	10,035	2,890	0.288	(	0.278	-	0.299	)	0.75	2.00	6.00
** **	75–79	3,339	7,706	1,790	0.232	(	0.222	-	0.243	)	0.75	2.75	-
** **	80–84	2,229	5,294	905	0.171	(	0.160	-	0.182	)	1.00	4.75	-
** **	85–89	1,097	2,338	322	0.138	(	0.123	-	0.154	)	1.75	6.75	-
	90+	639	1,163	119	0.102	(	0.086	-	0.123	)	3.25	-	-
**Number of**	0	5,836	10,347	3,234	0.313	(	0.302	-	0.324	)	0.50	2.00	6.75
comorbid diseases*	1–2	14,220	26,023	7,776	0.299	(	0.292	-	0.306	)	0.50	2.00	5.75
** **	3–5	10,604	20,117	4,825	0.240	(	0.233	-	0.247	)	0.75	2.50	7.25
** **	6 and more	1,915	3,236	630	0.195	(	0.180	-	0.210	)	1.00	3.00	8.50
**Severity of**	Low	22,084	41,884	9,909	0.237	(	0.232	-	0.241	)	0.75	2.75	8.75
type 2 diabetes*	Moderate	10,433	13,116	5,047	0.385	(	0.374	-	0.396	)	0.50	1.25	3.75
** **	High	3,274	4,647	1,509	0.325	(	0.309	-	0.342	)	0.50	1.50	4.50
Disability level*	No disability	23,046	48,338	15,127	0.313	(	0.308	-	0.318	)	0.50	1.75	5.25
** **	Disability level 1	3,094	5,039	765	0.152	(	0.141	-	0.163	)	1.25	4.75	-
** **	Disability level 2	2,877	4,584	463	0.101	(	0.092	-	0.111	)	2.50	8.75	-
** **	Disability level 3 (most severe)	1,154	1,717	110	0.064	(	0.053	-	0.077	)	4.00	-	-
**Participation in a**	No	25,280	56,196	14,571	0.259	(	0.255	-	0.264	)	0.75	2.25	7.25
type 2 diabetes DMP*	Yes	2,787	3,527	1,894	0.537	(	0.513	-	0.562	)	0.25	0.75	2.00
Total*		26,560	59,723	16,465	0.276	(	0.272	-	0.280	)	0.75	2.25	6.5

*Note: Number of comorbid diseases, severity of type 2 diabetes, disability level, and participation in a type 2 diabetes DMP are time-varying attributes. Persons with a change in an attribute are counted as persons with a specific time at risk in the former and later category of particular attribute. Thus, the time at risk and persons for these factors do not sum up to the values in "Total". Observation time starts at first valid type 2 diabetes diagnosis.

### Time to first visit after type 2 diabetes diagnosis

Overall, half of the persons in the sample had accessed an ophthalmologist within 2.25 years from first diagnosis (median KM-estimator, Table 2) while 18.5% of the persons who were newly diagnosed during the first observation period (third quarter of 2004) did not access an ophthalmologist at any time during the entire observation period (9.5 years until end of 2013). The median time to first visit was equal in both women and men indicated by a non-significant disparity (2.0 years for women and 2.25 years for men, log-rank test for equality: p = 0.20). The delay between diagnosis of type 2 diabetes and a first visit to an ophthalmologist increased with age from a median of 1.75 years in those aged 50–59 to 6.75 years in those aged 85–89 (log-rank test for trend: p<0.01). The greater the number of comorbidities, the longer the median delay before first accessing an ophthalmologist (log-rank test for trend: p<0.01). Conversely, with increasing severity of type 2 diabetes the delay in accessing an ophthalmologist decreased, with moderate and high severity type 2 diabetes patients accessing an ophthalmologist earlier compared to those with low severity (median 1.25, 1.50 and 2.75 years respectively (log rank test for trend: p<0.01). The delay to access an ophthalmologist was highly associated with level of disability. The median time to first visit increased from 1.75 years from diagnosis for those with no disability, to 8.75 years for those with disability level 2. Less than 50% of the type 2 diabetes patients with disability level 3 saw an ophthalmologist within the observation period (log-rank test: for trend: p<0.01). There was also a significant association between participation in a type 2 diabetes DMP and time to first visit. While 50% of the DMP participants had seen an ophthalmologist within nine months of diagnosis, median time from diagnosis to visit was 2.25 years for non-participants (log-rank test for equality: p<0.01).

### Time until first visit after type 2 diabetes diagnosis

On average, after adjusting for all covariates, women were more likely to visiting an ophthalmologist following diagnosis than men (hazard ratio (HR) 1.12, 95% CI 1.08–1.15; Table 3). The likelihood of visiting an ophthalmologist following diagnosis decreased with increasing age at diagnosis. When adjusting for all other covariates—especially for age and level of disability—persons with six or more comorbidities had a 26% higher chance of accessing an ophthalmologist following diagnosis compared to those with no comorbidities (95% CI 15%-37%). Increasing severity of type 2 diabetes was also associated with a greater chance of accessing an ophthalmologist (51% increase with moderate [95% CI 46%-56%] and 53% increase with severe type 2 diabetes [95% CI 45%-61%]). Conversely, with increasing levels of disability, persons had a lower chance of accessing an ophthalmologist, with a 41% decrease with disability level 1 [95% CI 36%-45%], a 58% decrease with disability level 2 [95% CI 54%-62%] and a 70% decrease with disability level 3 [95% CI 64%-75%] compared to those with no disability. Participants of a type 2 diabetes DMP had a 78% (95% CI 69%-87%) higher chance of seeing an ophthalmologist than the non-participants.

**Table 3:** Multivariate Cox regression analysis for time from diagnosis of type 2 diabetes to first visit to an ophthalmologist, third quarter of 2004 to last quarter of 2013, AOK data.

Characteristics		Hazard Ratio	p-Value	95%-Confidence interval				
**Sex**	Men	**1.00**						
** **	Women	1.12	*<0*.*01*	(	1.08	-	1.15	)
**Age at**	50–69	**1.00**						
**first diagnosis**	70–74	0.93	*<0*.*01*	(	0.89	-	0.97	)
** **	75–79	0.81	*<0*.*01*	(	0.77	-	0.86	)
** **	80–84	0.66	*<0*.*01*	(	0.62	-	0.71	)
** **	85–89	0.60	*<0*.*01*	(	0.53	-	0.67	)
** **	90+	0.50	*<0*.*01*	(	0.42	-	0.60	)
**Number of**	0	**1.00**	* *					
**comorbid diseases**	1–2	1.08	*<0*.*01*	(	1.03	-	1.12	)
** **	3–5	1.12	*<0*.*01*	(	1.07	-	1.17	)
** **	6 and more	1.26	*<0*.*01*	(	1.15	-	1.37	)
**Severity of**	Low	**1.00**	* *					
**type 2 diabetes**	Moderate	1.51	*<0*.*01*	(	1.46	-	1.56	)
** **	High	1.53	*<0*.*01*	(	1.45	-	1.61	)
**Disability level**	No disability	**1.00**	* *					
** **	Disability level 1	0.59	*<0*.*01*	(	0.55	-	0.64	)
** **	Disability level 2	0.42	*<0*.*01*	(	0.38	-	0.46	)
** **	Disability level 3 (most severe)	0.30	*<0*.*01*	(	0.25	-	0.36	)
**Participation in a**	No	**1.00**	* *					
**type 2 diabetes DMP**	Yes	1.78	*<0*.*01*	(	1.69	-	1.87	)

Note: Observation time starts at first valid type 2 diabetes diagnosis

The sensitivity analyses showed that the effects were highly robust (see S1 and S2 Tables). Only the effect of the number of comorbid diseases disappeared after the exclusion of the disability level. This was an indication for an interfering effect of disability level and number of comorbid diseases. Thus, the best model had to be adjusted for both covariates.

### Effect modification by sex

Further analyses revealed effect modifications of age (Likelihood-Ratio test: p = 0.04), severity of type 2 diabetes (Likelihood-Ratio test: p<0.01), and disability level (Likelihood-Ratio test: p<0.01) by sex, while for the number of comorbid diseases (Likelihood-Ratio test: p = 0.08) and for the participation in a type 2 diabetes DMP (Likelihood-Ratio test: p = 0.08) there were no significant improvements in the model fit (Table 4, see also S3–S7 Tables).

**Table 4:** Interaction effects of sex with the other covariates at first diagnosis in Cox regression analysis for time from diagnosis of type 2 diabetes to first visit to an ophthalmologist, results of five particular models with adjustments for the remaining covariates, third quarter of 2004 to last quarter of 2013, AOK data.

			Men							Women							Likelihood-Ratio Test
	Characteristics		Hazard Ratio	p-Value	95%-Confidence interval					Hazard Ratio	p-Value	95%-Confidence interval					
**Interactionmodel I**	**Age at first diagnosis**	50–69	**1.00**							**1.00**							11.56
		70–74	0.95	*0*.*10*	(	0.90	-	1.01	)	0.90	*<0*.*01*	(	0.85	-	0.96	)	p = 0.04
		75–79	0.84	*<0*.*01*	(	0.78	-	0.91	)	0.79	*<0*.*01*	(	0.74	-	0.84	)	
		80–84	0.72	*<0*.*01*	(	0.64	-	0.82	)	0.63	*<0*.*01*	(	0.58	-	0.69	)	
		85–89	0.73	*0*.*01*	(	0.57	-	0.92	)	0.56	*<0*.*01*	(	0.49	-	0.64	)	
		90+	0.73	*0*.*11*	(	0.50	-	1.08	)	0.45	*<0*.*01*	(	0.37		0.56	)	
**Interaction model II**	**Severity of type 2 diabetes**	Low	**1.00**							**1.00**							17.41
		Moderate	1.59	*<0*.*01*	(	1.52	-	1.67	)	1.43	*<0*.*01*	(	1.36	-	1.50	)	p<0.01
		High	1.68	*<0*.*01*	(	1.56	-	1.81	)	1.40	*<0*.*01*	(	1.29	-	1.51	)	
**Interactionmodel III**	**Disability level**	No disability	**1.00**							**1.00**							10.93
		Disability level 1	0.59	*<0*.*01*	(	0.52	-	0.67	)	0.59	*<0*.*01*	(	0.54	-	0.65	)	p = 0.01
		Disability level 2	0.50	*<0*.*01*	(	0.43	-	0.58	)	0.38	*<0*.*01*	(	0.34	-	0.43	)	
		Disability level 3 (most severe)	0.40	*<0*.*01*	(	0.29	-	0.55	)	0.26	*<0*.*01*	(	0.21	-	0.33	)	
**Interactionmodel IV**	**Number of comorbid diseases**	0	**1.00**							**1.00**							6.78
		1–2	1.10	*<0*.*01*	(	1.03	-	1.16	)	1.06	*0*.*04*	(	1.00	-	1.12	)	p = 0.08
		3–5	1.16	*<0*.*01*	(	1.09	-	1.24	)	1.08	*0*.*02*	(	1.01	-	1.15	)	
		6 and more	1.37	*<0*.*01*	(	1.22	-	1.54	)	1.13	*0*.*08*	(	0.99	-	1.29	)	
**Interaction model V**	**Partici-pation in a type 2 diabetes DMP**	No	**1.00**							**1.00**							3.09
		Yes	1.86	*<0*.*01*	(	1.73	-	1.99	)	1.70	<0.01	(	1.59	-	1.82	)	p = 0.08

Note: Sex-specific hazard ratios of the five covariates (age, severity, disability level, number of comorbid diseases, and DMP participation) are estimated in five separated models, hazard ratios of the particularly not in the interaction effect included covariates are omitted in the table

In case of age, there was a significant decrease of the chance of accessing an ophthalmologist in men up to the ages 80–89 (28% lower risk [95%-CI 18% - 36%]) compared to the men aged 50–69, and an increase in the likelihood for men at the highest ages. In contrast, there was a continuous and steep decrease in women: the lowest chance of assessing an ophthalmologist was observed for women at age 90+ (55% lower chance [95%-CI 44%-63%] compared to women at age 50–69).

As mentioned above, a high severity of type 2 diabetes increased the chance of visiting an ophthalmologist. The stratification by the sexes showed that the effect of a high severity of type 2 diabetes was much lower in women than in men. For example, men with a high severity of type 2 diabetes had a 68% [95%-CI 56%-81%] higher chance of a first visit compared to men with a low severity, while women with a severe type 2 diabetes had a 40% [95%-CI 29%-51%] higher chance.

A higher disability level had more impact in women than men. For example, the chance of a visit was 74% [95%-CI 67%-79%] lower for women with disability level 3 compared to women with no disability, while it was 60% [95%-CI 45%-71%] lower for men with disability level 3 compared to their counterparts with no disability.

In summary, of the adverse effects of age, disability level and severity of type 2 diabetes were worse in women than in men.

## Conclusions

In this study we demonstrated that half of all incident cases of type 2 diabetes have not had any DR screening within two years of their type 2 diabetes diagnosis and visits to an ophthalmologist were generally few in persons with type 2 diabetes. Older age, male sex and disability further impeded access to DR screening. This is not in keeping with current German guidelines which recommend annual visits in most persons with diabetes type 2 and potentially puts persons with type 2 diabetes at risk of unnecessary vision loss.

Our findings support those of several other studies which found that a large proportion of diabetic patients are not being screened for DR, or are having examinations far less frequently than the recommended interval. Estimates range between 20%-50% of patients who had annual check-ups and received DR management according to guidelines [27–29], and 35%-40% of physicians reported to not comply with guidelines for ophthalmological referral [30; 31]. Our findings were in keeping with this and indicated a considerable delay in accessing DR screening following a type 2 diabetes diagnosis.

In particular older patients and patients with disability had a lower rate of accessing DR screening in our study. In smaller samples including younger patients with type 2 diabetes, being younger (often <40 years) was associated with lower rates of DR screening [28; 32]. In another study of a comparable size to ours (n>50,000) both younger (≤34 years) and older (≥85 years) individuals were less likely to access DR screening [33]. As no persons below the age of 50 were included in our study we cannot assess how younger age might be associated with DR screening uptake. In older type 2 diabetes patients (50+) as represented in our study, increasing frailty and comorbidities as well as disability seem to play an important role in reducing access to DR screening and presumably other preventative interventions related to type 2 diabetes. In fact, a nationally representative study in South Korea found uptake of screening for DR to be associated with uptake of screening for microalbuminuria/nephropathy, and both to be impacted by low self-reported health status, which is in line with our findings [34]. All cited prospective studies had a follow-up much shorter than ours, ranging between 1.5 to 2 years [20–22], with one study assessing DR screening uptake retrospectively since disease onset in young adults over on average 13 years [35].

Receiving structured care as part of a type 2 diabetes DMP considerably increased the chance of accessing DR screening in our study. This reflects other studies’ findings of better access to screening and referral systems within type 2 diabetes DMPs. Several studies reported increased numbers of eye examinations as well as a higher proportion of patients accessing DR screening [24; 36–38]. Enrolling more patients shortly after the first type 2 diabetes diagnosis into structured care plans such as DMPs might considerably improve care indicators such as regular DR screenings and thus preserve vision. Alternatively, new models of service provision with DR screening in primary practice utilizing telemedicine platforms might circumvent some barriers in accessing ophthalmic DR screening. In a recent study, retinal telescreening implemented in a primary care setting was shown to increase DR screening uptake [39; 40].

This study has a number of strengths. Notably, the diagnoses of the type 2 diabetes, chronic eye diseases and severe comorbidities were based on diagnoses coded by professional physicians and validated by a confirming diagnosis. Thus, validity of the medical information can be assumed to be high. The data used are provided by Germany’s largest health insurance provider (AOK), which covers one third of the population. Since a large random sample was the basis for the analysis, the results are highly representative for the AOK population. However, the AOK members are on average older and unhealthier than the overall German population [41; 42]. Thus, our results need to be interpreted with caution in relation to the overall German population. The prospective cohort design allows for the assessment of temporal trends in health and healthcare utilization over a long period of time. In contrast to data collected from healthcare providers directly, health claims data are independent from the choice of the physician, hospital and place of residence. The bias due to self-selected dropouts is minimal compared to the bias in survey-based studies as data are captured irrespective of whether patients move or change health providers. Also, our sample includes the complete population living in the community, both in private households and institutions.

There are some limitations such as the definition of persons under study. The analysis is focused on persons aged 50+ with incident type 2 diabetes and no diagnosis of a chronic eye disease preceding the type 2 diabetes diagnosis. With increasing age this group is increasingly more selective. Further analyses should include younger age groups to assess disparities within these subgroups. Data from health insurance providers in other countries may be biased towards those with greater financial means, however that is unlikely to be important in this study because health insurance is compulsory for all German residents.

Another limitation is the definition of the outcome variable “visit to an ophthalmologist”, which is defined by at least one diagnosis reported by an ophthalmologist and considered as an indicator for DR screening. Visits without any reported diagnoses are not registered in the accounting data. However, since non-reporting of a diagnosis will lead to no reimbursement for the health provider, it is likely that all visits to an ophthalmologist are accurately captured. DR screening rates may therefore have been overestimated. However, the impact of any overestimation is likely to be minimal because only persons who had not been regularly visiting an ophthalmologist at the time of type 2 diabetes diagnosis were included in this study, and it is reasonable to assume that DR screening took place. Conversely, patients might have been included in whom a chronic eye disease, such as cataract or retinopathy potentially associated with undiagnosed type 2 diabetes, was diagnosed in the same quarter as the type 2 diabetes. This would lead to an overestimation of the incidence of a first visit to an ophthalmologist in the quarter of first type 2 diabetes diagnosis. In order to assess this, we excluded all patients (n = 1,740) with a coincidental first visit to an ophthalmologist and type 2 diabetes diagnosis in one quarter in a sensitivity analysis. This did not change the inference of the results and can thus be disregarded as a source of bias. We lack information about the health status of patients prior to 2004 which is known as left truncation. It causes bias in cases of a low willingness or ability to attend regular medical check-ups. These persons may falsely be defined as incident type 2 diabetes cases or as healthy.

Participation in a DMP may be influenced by self-selection of persons (for example more health-conscious persons) or selection by physicians (for example more unhealthy persons). We have no information on the DMP enrolment process, but it is unlikely that this limitation much impacts our findings as we controlled for number of comorbidities, severity of type 2 diabetes and present disability. Finally, we have no data on socio-economic factors, knowledge related to type 2 diabetes, health literacy or residence which have all been shown to impact DR screening uptake. However, given our sample size, the associations we found are unlikely to be confounded by either.

In conclusion, the majority of newly diagnosed type 2 diabetes patients did not meet currently recommended goals for DR screening. They are therefore at greater risk of missing out on opportunities to maintain good vision. Screening rates were particularly low among older patients, men, and patients with a disability. Being enrolled in a DMP improved DR screening uptake considerably suggesting that increased participation in these programs may lead to improved overall screening uptake. Our study has implications for the provision of DR screening for type 2 diabetes which needs to be improved to reach patients in need.

## Supporting information

S1 Table Sensitivity analyses: Comparison complete model with models excluding covariates (part one), AOK data. (DOCX)

S2 Table Sensitivity analyses: Comparison complete model with models excluding covariates (part two), AOK data. (DOCX)

S3 Table Results of the Cox regression model with the interaction effect of sex and age at first diagnosis, adjusted for severity of type 2 diabetes, disability level, number of comorbid diseases, and DMP participation, AOK data. (DOCX)

S4 Table Results of the Cox regression model with the interaction effect of sex and severity of type 2 diabetes, adjusted for age at first diagnosis, disability level, number of comorbid diseases, and DMP participation, AOK data. (DOCX)

S5 Table Results of the Cox regression model with the interaction effect of sex and disability level, adjusted for age at first diagnosis, severity of type 2 diabetes, number of comorbid diseases, and DMP participation, AOK data. (DOCX)

S6 Table Results of the Cox regression model with the interaction effect of sex and number of comorbid diseases, adjusted for age at first diagnosis, severity of type 2 diabetes, disability level, and DMP participation, AOK data. (DOCX)

S7 Table Results of the Cox regression model with the interaction effect of sex and DMP participation, adjusted for age at first diagnosis, severity of type 2 diabetes, disability level, and number of comorbid diseases, AOK data. (DOCX)
