# Supplementary material for: Diabetic retinopathy screening in incident diabetes mellitus type 2 in Germany between 2004 and 2013 - A prospective cohort study based on health claims data
Source: PLoS One. 2018 Apr 5;13(4):e0195426. doi: 10.1371/journal.pone.0195426 (PMC5886553; doi:10.1371/journal.pone.0195426)
Supplement: S3 Table — (DOCX) [file pone.0195426.s003.docx]

S3 Table: Results of the Cox regression model with the interaction effect of sex and age at first diagnosis, adjusted for severity of type 2 diabetes, disability level, number of comorbid diseases, and DMP participation, AOK data
