# Supplementary material for: Diabetic retinopathy screening in incident diabetes mellitus type 2 in Germany between 2004 and 2013 - A prospective cohort study based on health claims data
Source: PLoS One. 2018 Apr 5;13(4):e0195426. doi: 10.1371/journal.pone.0195426 (PMC5886553; doi:10.1371/journal.pone.0195426)
Supplement: S7 Table — (DOCX) [file pone.0195426.s007.docx]

S7 Table: Results of the Cox regression model with the interaction effect of sex and DMP participation, adjusted for age at first diagnosis, severity of type 2 diabetes, disability level, and number of comorbid diseases, AOK data
